# Comparative assessment of tissue cross-reactivity and pharmacokinetic half-life of malaria monoclonal antibodies

**DOI:** 10.3389/fimmu.2026.1848315

**Published:** 2026-06-23

**Authors:** William R. Fugina, Dallas R. Brown, Shelby Foor, Emma C. Ryan, Samuel Cuevas, Shreeram Nallar, Dawn Wolf, Renita Brown, Jesse P. Deluca, Geoffrey C. Chin, Elizabeth J. Raymond, James T. Raymond, Phil S. Medlin, Sheetij Dutta

**Affiliations:** 1 In vivo Services Branch, Center for Enabling Capabilities, Walter Reed Army Institute of Research, Silver Spring, MD, United States; 2 Structural Vaccinology Laboratory, Biologics Research and Development Branch, Center for Infectious Disease Research, Walter Reed Army Institute of Research, Silver Spring, MD, United States; 3 Experimental Therapeutics Branch, Center for Infectious Disease Research, Walter Reed Army Institute of Research, Silver Spring, MD, United States; 4 Immunopathology, Charles River Laboratories, Reno, NV, United States; 5 Pathology Branch, Center for Enabling Capabilities, Walter Reed Army Institute of Research, Silver Spring, MD, United States

**Keywords:** circumsporozoite protein, mAb 311, mAb 317, malaria, monoclonal antibody (mAb), rhesus macaque, tissue cross-reactivity

## Abstract

**Introduction:**

Monoclonal antibodies (mAbs) targeting the *Plasmodium falciparum* circumsporozoite protein (CSP) are leading clinical candidates for malaria prevention in travelers, military personnel, and pregnant women. Off-target binding and pharmacokinetics (PK) are important considerations during hit-to-lead optimization. As part of a programmatic pivot toward developing malaria mAb candidates, a human tissue cross-reactivity (TCR) and half-life extension (HLE) method optimization was performed using prototype *P. falciparum* mAbs.

**Methods:**

TCR assay was performed across 14 human tissue types using the CSP mAbs 317 and 311 and the clinical reference mAb CIS43. The HLE optimization was performed on mAb 311 by introducing Fc modifications that improve affinity for human neonatal Fc receptor (FcRn). Safety and pharmacokinetic parameters of the modified mAb 311-LS and the wild-type 311-WT was determined by intravenous administration in the rhesus PK model at 10 mg/kg dose. Concentration of the mAbs was measured using enzyme-linked immunosorbent assay and biolayer interferometry.

**Results:**

TCR assay showed no membrane binding, but the major repeat-specific mAbs 317 and 311 exhibited varying degrees of positive cross-reactivity to extracellular and cytoplasmic elements in multiple tissue types. In contrast, the clinical reference mAb CIS43 (junctional epitope) showed no TCR positivity. The prototype mAb 311 binding affinity to human neonatal Fc receptor (FcRN) was significantly improved by introducing commonly known HLE mutations. MAb 311-LS was found safe and showed an improved pharmacokinetic profile compared to the wild-type mAb 311.

**Discussion:**

These findings highlight the critical need to evaluate TCR liabilities early in the mAb process development cycle. The successful improvement of mAb 311-LS half-life demonstrates that Rhesus is an excellent model to compare HLE strategies. The developability benchmarks described here will help in the translation of novel, long-acting anti-malarial mAbs.

## Introduction

The World Health Organization reported 282 million cases and 610,000 deaths globally due to *Plasmodium falciparum* malaria in 2024 (1). Despite access to prophylactic regimens, 30 cases of malaria were reported among United States service members in 2024 (2). Two malaria vaccines, RTS,S/AS01 and R21/Matrix M, are approved for use in pediatric populations in Sub-Saharan Africa (3), but are yet to be approved for highly susceptible adult populations, including pregnant women, immunologically naïve tourists, and military personnel. As insecticide resistance in mosquitoes and drug resistance among parasite strains threaten global malaria control programs (4), more durably protective vaccines (5–7), single-dose injectable regimens of monoclonal antibodies (mAbs) (8), and long-acting chemoprophylactic agents (9) are under consideration as additional tools to achieve malaria elimination targets.

MAbs have been approved for the prevention and control of infectious diseases including respiratory syncytial virus (RSV) (10, 11), SARS-CoV-2 (12, 13), Ebola virus (14), *Clostridioides difficile* (15), anthrax (16), and HIV (17, 18). *P. falciparum* circumsporozoite protein (CSP), the antigen used in both RTS,S and R21 vaccines, is also targeted by the most advanced clinical mAb candidates against malaria. MAb CIS43 was isolated from an attenuated whole sporozoite vaccine recipient (19–22) and binds to the conserved junctional repeat (NPDP). In controlled human malaria infection (CHMI) trials, 5, 20, and 40 mg/kg of CIS43 were administered intravenously (IV), conferring sterilizing protection against mosquito bite challenge at serum concentrations ranging from 46 to 493 µg/mL (19). In phase 2b trials in Mali, 10 and 40 mg/kg of CIS43 (IV) demonstrated 75% and 88% efficacy (time to first infection), respectively (20, 23). MAb CIS43, titrated between 1 and 10 mg/kg (IV and SC), showed 57% sterile protection in higher dose groups at 10–15 µg/mL serum concentration. Subjects with >33 µg/mL CIS43 in the serum were fully protective (21). Another malaria mAb candidate L9, isolated from an attenuated sporozoite vaccine recipient, binds to the minor repeat (NPNV) epitope (24). In CHMI trials, mAb L9 at 1–20 mg/kg dose (IV and SC) conferred 80%–100% protection in subjects with 9–388 µg/mL L9 serum concentration (25). In phase 2b trials in Malian children, L9 at 150- and 300-mg doses (SC) showed 66% and 70% efficacy (time to first infection), respectively, during the 6-month follow-up (26). CSP mAbs 311 and 317 that were isolated from RTS,S vaccinees (MAL071 trial) were the subject of extensive structure–function studies (27–29). Further down-selection resulted in the clinical candidate MAM01, targeting the NPNA major repeats, being developed and tested at the 1.5–40 mg/kg dose (SC and IV) in a CHMI trial (30). MAM01 conferred 0%–100% protection at 2.4–160 µg/mL serum concentration and >88 µg/mL MAM01 conferred sterile protection (31). The proof of concept of several passively infused mAbs conferring protection against malaria, at clinically sustainable concentrations, is now well established. A deployable candidate is likely to emerge based on superior developability criteria: *in vivo* pharmacokinetics (PK) [absorption, distribution, metabolism, and elimination (ADME)], immunogenicity [anti-drug antibody (ADA)], specificity and off-target effects, manufacturing yield, storage stability, acceptability of delivery route, and, most importantly, cost of goods.

Molecular mimicry and cross-reactivity of antibodies against *Helicobacter* (32), HIV (33), *Campylobacter* (34), and SARS-CoV-2 (35) are well known. Hence, minimal off-target binding of prophylactic antibodies is a developability criterion for clinical progression. In addition to *in vivo* animal toxicology, regulatory agencies accept *in vitro* surrogates such as human proteome array (36) and histological tissue cross-reactivity (TCR) (37), as evidence of preclinical safety prior to first-in-human trials (37–39).

The pharmacokinetic half-life (*t*_1/2_) of immunoglobulin G (IgG) in humans is ~20 days; thus, optimizing serum retention time is another key developability attribute for mAbs (40). Protein engineering strategies were implemented to enhance *t*_1/2_ for licensed mAb products, e.g., RSV Nirsevimab (*t*_1/2_ ~70 days) (10, 11), SARS CoV-2 Tixagevimab/Cilgavimab (*t*_1/2_ ~80 days) (12), and Sotrovimab (*t*_1/2_ ~50 days) (13, 41). IgG administered *in vivo* is pinocytosed by endothelial cells, macrophages, and dendritic cells and trafficked to the endosomal compartment for non-specific degradation. The neonatal Fc receptor (FcRn) present in the endosomal membrane can rescue IgG molecules by binding and recycling them back to the extracellular environment. At low endosomal pH, the CH3 and CH2 domain of IgG (residues I253, T254, H310, H433, and H435) interact with FcRn (residues E115 and D130) (42). Half-life extension (HLE) involves introducing Fc mutations that increase FcRn-Fc binding affinity by 2- to 40-fold (43–45). Successful HLE mutations in the Fc region of IgG include LS (44, 46, 47), YTE (45, 48, 49), DHS (50), QVV (51), and KF (52, 53).

As part of a programmatic pivot toward mAb development, our lab utilized structurally defined prototype CSP mAbs known to specifically inhibit malaria sporozoite invasion into mammalian liver cells. We previously reported correlations between *in vitro* and *in vivo* assays for early preclinical down-selection of novel mAbs (54). Here, we present the results of two developability assays using prototype CSP mAbs 317 and 311, with these data serving as benchmark for prophylactic mAb development against malaria.

## Materials and methods

### Expression and characterization of mAbs

Wild-type mAbs 317, 311, and CIS43 were expressed and purified by a commercial entity (Atum, Newark, CA) (54). HLE mutations (**Supplementary Figure 1**) M450L/N456S (LS) (44, 46, 47), M274Y/S276T/T278E (YTE) (45, 48, 49), L331D/Q333H/N456S (DHS) (50), T329Q/Q333V/A400V (QVV), or H455K/N456F (KF) (52, 53) were introduced into the heavy chain of mAb 311, and the corresponding DNA was produced using the Qiagen EndoFree Maxiprep kit. Transfections were carried out at the 200-mL scale using the Expi293 Mammalian Expression System (ThermoFisher). IgG was purified using Protein-G Sepharose (Pierce) columns at a 2-mL scale per the manufacturer’s recommendations. Purity was assessed by SDS-PAGE under reduced and non-reduced conditions. Reactivity to the NANPx6 peptide was performed using enzyme-linked immunosorbent assay (ELISA) as described previously (54). Large-scale expressions of mAb 311-LS and 311-WT were done using Expi293 cells (ThermoFisher) per the manufacturer’s recommendations. Culture supernatant was harvested 1 week post-transfection, diluted 1:1 with IgG Binding Buffer (Thermo Fisher Scientific), and loaded onto a 10-mL Protein G Sepharose column mounted on an AKTAPure FPLC system (GE Healthcare, Chicago IL). The column was washed with 300 mL of IgG Binding Buffer, after which IgG Elution Buffer was applied. Fractions (50 mL) were collected and neutralized using Tris-HCl, and the final IgG prep was dialyzed against phosphate-buffered saline (PBS). Purity was measured by SDS-PAGE and an endotoxin assay was performed using *Limulus* amebocyte lysate (Associates of Cape Cod, Falmouth MA). Nano DSC (TA Instruments, New Castle DE) was used to measure thermal transitions. The DSC system baseline was collected using degassed buffer (PBS), and reference and sample cells were injected with 600 µL of degassed buffer or antibodies at 4 mg/mL. The system was allowed to equilibrate for 10 min, and data were collected by heating at 1 °C/min (25–95 °C), followed by cooling at 2 °C/min for the same temperature range. Raw data were processed by NanoAnalyze software.

### Tissue cross-reactivity assay

A non-GLP TCR study was run according to Charles River Laboratory’s standard protocols. CSP mAbs 311, 317, and a laboratory preparation of the clinical candidate CSP mAb CIS43 were tested at 20 and 5 µg/mL. A polyclonal human IgG1κ antibody (HuIgG1) with no known antigenic specificity was used as the isotype control. Pellets of CSP-transfected HEK293 cells served as the positive control article and non-transfected cells served as the negative control. An assay control slide for each tissue was also produced to evaluate background staining associated with the immunohistochemical procedure. A polyclonal rabbit anti-β_2_-microglobulin antibody was used as the tissue staining control for all tissues to confirm their suitability for immunohistochemistry staining. The lower concentration of mAb in TCR assays was selected based on reproducible staining of the positive control CSP transfected cells by mAb 317 (5 μg/mL), and the higher concentration was four times of this optimal concentration (20 μg/mL). Cryosections (5 μm thick) of normal human tissues from adrenal gland, cerebellum, cerebrum, heart, kidney, liver, lung, muscle, ovary, pancreas, skin, small intestine, spleen, testis, and thyroid gland (one donor per tissue) were tested. Tissues were fixed in acetone and 10% neutral-buffered formalin, rinsed twice with TBS (Tris-buffered saline; pH 7.6), and incubated with the Dako peroxidase blocking reagent for 5 min to quench endogenous peroxidase. Tissues were again rinsed twice with TBS and incubated with the avidin solution for 15 min, rinsed once with TBS, incubated with a biotin solution for 15 min, and rinsed once with TBS. Tissues were treated with a protein block to reduce nonspecific binding for 20 min. A precomplexed primary and secondary antibody mix was applied to the slides for 2 h, after which slides were rinsed twice with TBS, treated with the ABC Elite reagent (Vector, Catalog No. PK-6100) for 30 min, rinsed twice with TBS, and then treated with DAB peroxidase substrate (Sigma, Catalog No. D4418) for 4 min. All slides were rinsed with tap water, counterstained, dehydrated, and mounted. After staining, slides were visualized under light microscopy by an immunopathologist. Each slide (test mAb, isotype control, or assay control) was examined for the presence of stained cell types or tissue elements. Each stained cell type and tissue element was identified, the subcellular (or extracellular) location of the staining was recorded, and the intensity (strength) of staining was assigned. Frequency of cell-type staining was also assigned to provide the approximate percentage of cells of a given cell type or tissue element with staining. The slides, once examined by CRL, were shipped to Walter Reed Army Institute of Research (WRAIR) for digital scanning and confirmatory review by an independent immunopathologist.

### FcRn binding using biolayer interferometry

Biolayer interferometry (BLI) was performed using the OctetRED96e instrument (Fortebio – Sartorius). FAB2G sensors were coated with 3 µg/mL 311-WT or 311-HLE for 60 s in 100 mM sodium phosphate, 150 mM NaCl, and 0.05% Tween-20 (pH 6.0). The baseline was established for 60 s, after which recombinant FcRn (Acro Biosystems, Newark DE) was associated at 92, 46, 23, 11.5, 5.75, 2.8, and 0 µg/mL for 60 s and dissociated for 120 s. Data Acquisition and Analysis Software Version 12.0 was used to determine association and dissociation rates and the equilibrium dissociation constant (*K*_D_).

### NANPx6 binding using BLI

Biotinylated NANPx6 peptide (0.1 µg/mL) was loaded on a Streptavidin Biosensor for 200 s in Kinetics Buffer (pH 7.4). The baseline was established for 200 s, after which mAb 311WT or 311-LS at 10 µg/mL was associated for 180 s and dissociated for 350 s. *K*_D_ values were calculated using a local (individual) fitting using a narrowed window framing of 30–85 s for the association step and 10–350 s for the dissociation step.

### Mouse protection

Female C57BL/6 mice (The Jackson Laboratory, Bar Harbor, ME) were housed at the WRAIR animal facility under an IACUC-approved protocol. All mice were confirmed negative for common laboratory mouse-associated pathogens. Mice were group-housed with same-sex conspecifics in ventilated microisolator cages and maintained on corncob bedding and standard rodent chow (LabDiet or equivalent) with water provided *ad libitum*. Environmental enrichment was provided in accordance with institutional animal care standards. Animals were maintained in temperature- and humidity-controlled rooms on a 12:12-h light–dark cycle in accordance with standard laboratory animal husbandry practices. Mice were administered 300 µg of mAb 311-WT, 311-LS dose, or sterile PBS (*n* = 5) by IV injection into the caudal tail vein. The 300-µg dose chosen was based on a previous report (47). Efficacy was evaluated 24 h post-injection with 1,000 transgenic *P. berghei* engineered to express full-length *P. falciparum* CSP (Wellcome strain) in 100 µL of sterile PBS administered via IV injection. Parasitemia was monitored daily by observing 10–20 fields of Giemsa-stained blood films starting 4 days post challenge (55). Mice were considered unprotected after 2 consecutive days of parasitemia, or counted as protected if no parasitemia was detected up to 2 weeks after challenge. Pairwise log rank test was performed on the survival curves.

### Rhesus

US-bred adult rhesus macaques of Indian origin, aged 4 years, were housed at the WRAIR animal facility under an IACUC-approved protocol. All macaques tested seronegative for macacine herpesvirus 1, measles, simian retrovirus, simian immunodeficiency virus, and simian T-cell leukemia virus, and were tuberculin skin test negative. They were also pre-screened for pre-existing antibodies against *P. falciparum* CSP. The animals were pair-housed with same-sex conspecifics, fed a commercial diet (Lab Diet 5038, Purina Mills International), and provided water *ad libitum*, along with a variety of fresh fruits and vegetables. Environmental enrichment was provided in accordance with standard practices, and automatic lighting was maintained on a 12:12-h cycle. To minimize the need for sedation and anesthesia, the macaques were acclimatized to a restraint table for blood collection, which was generally well tolerated with the use of food enrichment.

### Rhesus infusions

Four rhesus monkeys, two males and two females, were chosen and randomly assigned to two groups (*n* = 2 per group). Animals were injected with ketamine and dexmedetomidine intramuscularly in the caudal thigh. Once sedated, the saphenous vein was selected for catheter placement. The area was shaved, cleaned, and disinfected with a minimum of three alternating 70% isopropyl alcohol and chlorohexidine scrubs. A catheter was placed in the vein using aseptic technique and patency checked with the injection of sterile NaCl fluids. Once patency was confirmed, mAb 311-LS or 311-WT was dosed at 10 mg/kg IV based on a previous report (47). The mAb was transfused over a 1-min period followed by 1 mL of sterile NaCl to flush the catheter. Whole blood was collected (Vacutainer SST Tubes, Becton, Dickinson, Franklin Lakes, NJ), clotted, and spun at 1,300 RCF for 10 min at room temperature. Serum was aspirated and transferred to 2.0-mL cryovials (Corning) and stored at −80 °C for long-term storage or at 4 °C between assays.

### Safety and tolerability assessment

NHPs were closely monitored for signs of immediate hypersensitivity reaction, fever, redness, swelling, anaphylaxis, and cardiac and respiratory abnormalities while under sedation during the mAb infusion phase. Following mAb injection, animals were monitored for local injection site reactions, hypersensitivity, gastrointestinal symptoms, and general stress responses. Blood samples were taken at 0, 2, 4, and 6 h post-mAb infusion and then on days 1, 3, 6, and 7, followed by twice weekly (approximately 3–5 days apart) for up to 8 weeks, and then once a month up to 20 weeks.

### Quantification of mAb 311 concentration by ELISA

Immulon 4HBx ELISA plates (ThermoFisher, Hanover Park IL) were coated with full-length CSP protein (100 ng/well) overnight at 4 °C, washed 3× with PBS plus 0.05% Tween-20 (PBST), and blocked using Blocker Casein™ (Pierce) for 1 h in a humidity chamber. MAb 311-WT quantitation standards were included on each plate. The standard was produced at 0.5 µg/mL and diluted 3-fold in duplicate (0.5–0.002 µg/mL). As a plate control, 1:50 naïve serum pool was added to one well, and 150 µL of 1:50 dilution of unknown sera was serially diluted threefold. Plates were incubated at room temperature for 2 h in a humidity chamber, washed 3×, followed by incubation with 100 µL of 1:4,000 goat anti-human Ig-HRP (Southern Biotech) for 1 h. Plates were washed 4× and incubated with 100 µL of KPL ABTS Peroxidase Substrate (SeraCare, Milford MA) for 1 h in the dark, and the reaction was arrested using 10 µL of 20% SDS. Absorbance at 415 nm was recorded on a Synergy4™ plate reader (Agilent/Biotek, Santa Clara CA). Using the mAb standard, a 4-Parameter Logistic Curve was used to convert OD vs. dilution curve to OD vs. mAb concentration using non-linear regression (Gen5™ Software). Mean OD for the pre-bleed at 1:50 dilution for four animals indicated the assay background to be ~0.18 µg/mL (SD = 0.180) and the lower limit of quantitation ~2.5 µg/mL.

### Quantitation of mAb 311 by BLI

The OctetRed96*e* instrument was used to quantify 311-WT and 311-LS in rhesus serum. A 311-WT stock was diluted in naïve rhesus serum pool and a 1:1 dilution was used to produce the standard curve (1.75 to 0.10 µg/mL). Briefly, the HIS1K sensors were regenerated for 10 s in 20 mM glycine (pH 1.5), neutralized for 10 s in Kinetics Buffer, and loaded with 6×Histidine tagged recombinant CSP (0.65 µg/mL) for 180 s. The baseline was established for 150 s, then association with standard, pre-bleed, or the test sera at 1:300 dilution occurred for 300 s. The nm shift from 0.25% naïve serum pool was subtracted, and the linear regression (*R*^2^ values >0.98) value from the standard was used to convert BLI nm shift at 300 s to mAb concentration. The mAb standards were run at the beginning and the end of each run to control sensor performance, and the assay was replicated twice.

### Pharmacokinetic analysis

Phoenix^®^ PK/PD Software (WinNonlin version 8.6.1.6) was used to analyze the ELISA and BLI data. Non-compartmental analysis was used to estimate the elimination rate constant (*k_e_*), maximal concentration (*C*_max_), time to maximal concentration (*T*_max_), and time above therapeutic level of 50 μg/mL. The pharmacokinetic parameters terminal half-life (*t*_1/2_), area under the concentration–time curve extrapolated to infinity (*AUC*_∞_), apparent volume of distribution (*V_z_*), and apparent clearance (CL) were derived from *k_e_*. Concentration–time profiles were generated for visual comparison.

### Anti-drug antibody detection

Rhesus serum on days 0, 56, 87, 94, 104, 111, 132, 139, 193, and 200 post-infusion was evaluated for the presence of mAb 311-binding antibodies using BLI. Streptavidin biosensors (Sartorius, 18-5020) were loaded with biotinylated mAb-311 at 2.5 µg/mL for 300 s. The baseline was established for 60 s, then rhesus sera (1:50 dilution in Kinetics Buffer) were associated for 300 s. Goat anti-rhesus IgG (Southern Biotech, Birmingham AL) was used to plot a standard curve. All runs were normalized to a blank reference sensor, and binding of antibodies to mAb 311 was plotted following the 300-s association.

## Results

### Tissue cross-reactivity

**Figure 1:**
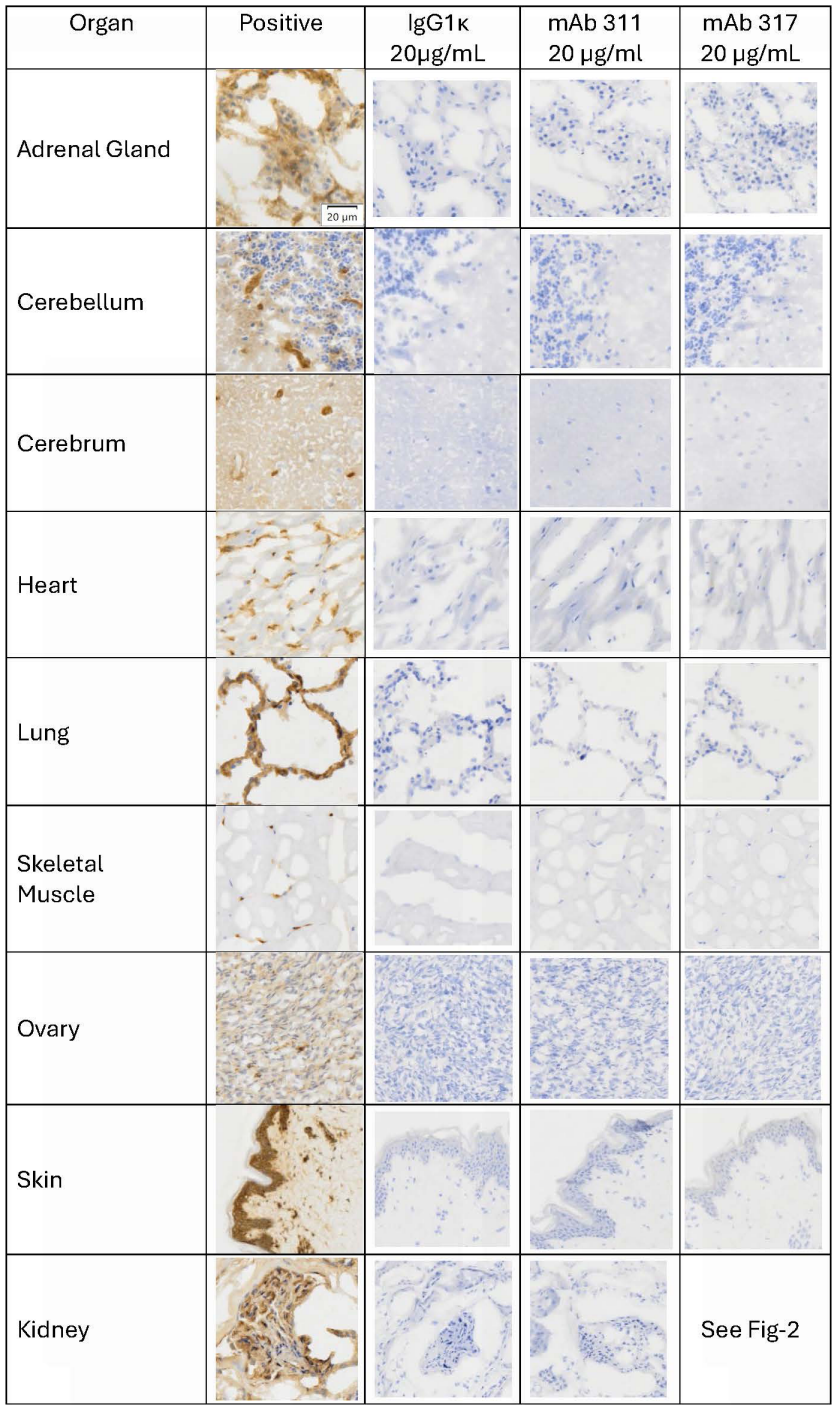
Negative tissue cross-reactivity of mAb 311 and 317 against nine tissue types. Immunoreactivity profile of 20 µg/mL IgG1κ (negative control), 20 µg/mL mAb 311, or 20 µg/mL mAb 317. The kidney panel for mAb 317 was left blank as it reacted positively (**Figure 2**).

**Figure 2:**
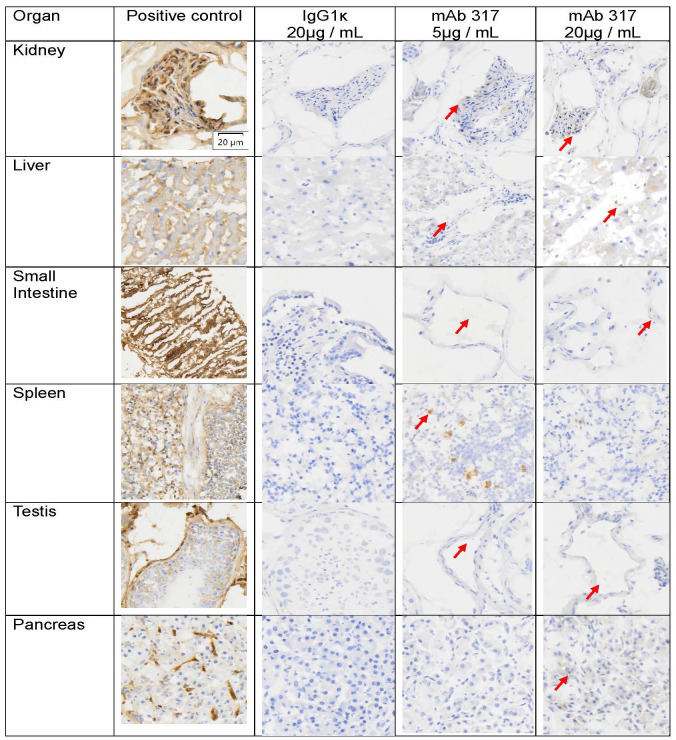
Positive reactivity of mAb 317 against six human tissue types. Beta 2-microglobulin (positive control), IgG1k (negative control), and mAb 317 tested at two concentrations. MAb 317 was found to have extracellular reactivity to granular material of the kidneys (glomerular tufts) and cytoplasmic reactivity within the tubular epithelium. There was cytoplasmic reactivity in liver platelets within blood vessels, platelets of the small intestine, splenic mononuclear cells, and in the acinar epithelium within the pancreas.

**Figure 3:**
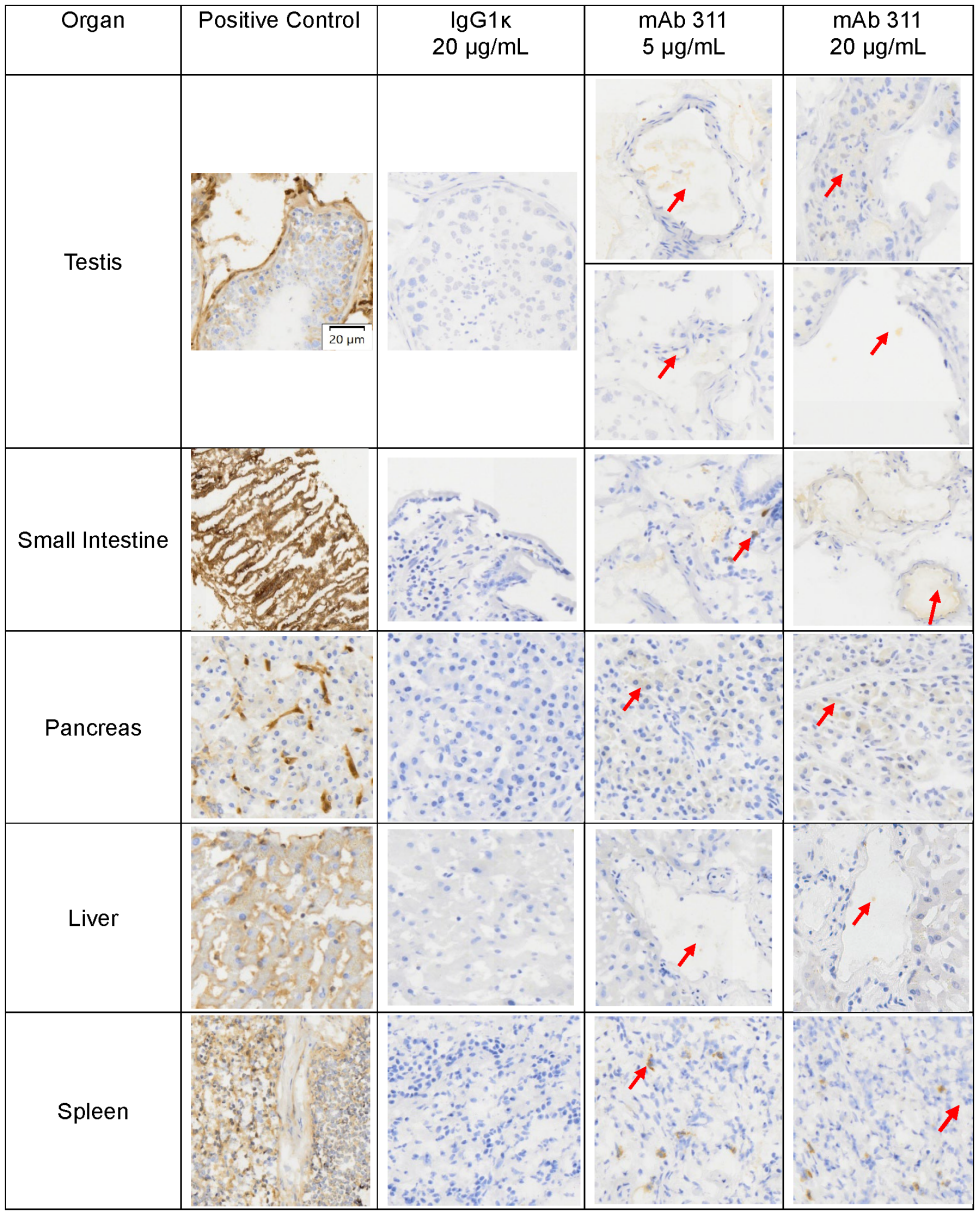
Positive reactivity of mAb 311 against five human tissue types: Beta 2-microglobulin (positive control), IgG1k (negative control), and mAb 311 were tested at two concentrations. Immunoreactivity with extracellular intra- and extravascular granular material of the testis and weak cytoplasmic immunoreactivity of spermatogenic epithelium (Testis top panel) and platelets within testicular blood vessels (Testis bottom panel). There was positive reactivity with small intestinal intra- and extravascular granular extracellular material. Cytoplasmic reactivity occurred within the acinar epithelium of the pancreas, platelets within blood vessels of the liver, and in splenic mononuclear cells.

MAbs were applied to cryosections of 14 selected normal human tissues (one donor per tissue) at 20 and 5 µg/mL. Prototype mAbs 311 and 317 (major repeat epitope) were evaluated along with the reference mAb CIS43 (junctional epitope) to ensure assay specificity. The positive control article, CSP-transfected HEK293 cells, showed strong membranous and cytoplasmic immunoreactivity with mAb 311 and 317, while no immunoreactivity against mock-transfected cells was noted (**Supplementary Figure 2**). Polyclonal rabbit anti-β_2_-microglobulin revealed intense staining across tissues, while human IgG1κ isotype control showed no reactivity (**Figures 1**–**3**). No cell membrane immunoreactivity was noted for any of the CSP mAbs across the 14 assayed tissue types. The reference mAb CIS43 showed no extracellular or cytoplasmic reactivity against any of the tissues examined (**Supplementary Figure 3**). While mAb 317 tested negative for binding to eight human tissue types (**Figure 1**), extracellular and cytoplasmic reactivities against six tissue types (**Figure 2**) were observed. Specifically, mAb 317 immuno-reacted with extracellular, granular material in glomerular tufts of the kidney and had cytoplasmic immunoreactivity within the tubular epithelium of the kidney (**Figure 2**). MAb 317 exhibited positive cytoplasmic immunoreactivity within platelets in the liver, small intestine, and testis; within mononuclear cells in the spleen; and within the acinar epithelium of the pancreas. Likewise, the mAb 311 showed no membrane reactivity and no cytoplasmic or extracellular reactivity in nine tissue types (**Figure 1**). Five tissues showed weak immunoreactivity with mAb 311 (**Figure 3**); these included extracellular granular material in the testis and small intestine (**Figure 3**, top testis panel) and cytoplasmic material in the acinar epithelial cells of the pancreas, as well as platelets in the liver and germinal epithelial cells, including Sertoli cells, spermatogonia in the testis (**Figure 3**, bottom testis panel), and mononuclear cells in the spleen.

### Half-life extension strategies

**Figure 4:**
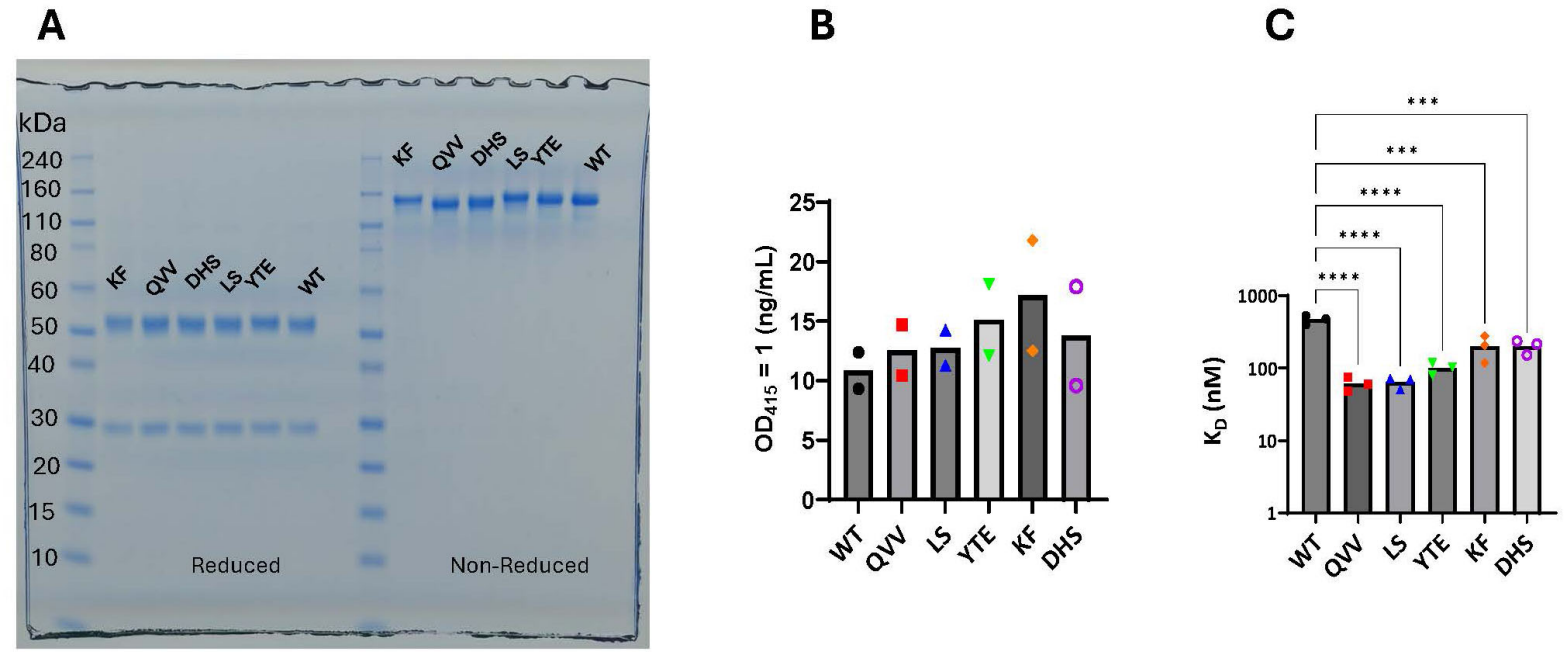
Characterization of Fc-modified mAb 311 IgG. Antibodies containing Fc mutations in the heavy-chain Fc region: T307Q/Q311V/A378V (QVV), M451L/N457S (LS), M252Y/S254T/T256E (YTE), H433K/N434F (KF), and L309D/Q311H/N434S (DHS). **(A)** SDS-PAGE Coomassie blue-stained reduced and non-reduced gel. **(B)** Binding of Fc variants to NANPx6 peptide measured by ELISA. Mean mAb concentration for OD_405_ = 1 (duplicate wells). **(C)** Mean dissociation constant (*K*_D_) for Fc variant binding to human FcRN at pH 6.0 measured by BLI (triplicate run).

The observed reactivity of mAb 317 with the kidneys excluded its progression toward HLE optimization. We therefore proceeded with the prototype mAb 311 for HLE engineering. Unengineered (WT), LS, YTE, DHS, QVV, and KF heavy-chain Fc mutations were introduced in mAb 311 and expressed in HEK293 cells (**Figure 4A**). The HLE variants bound to the NANPx6 peptide (<20 ng/mL resulting in OD = 1) by ELISA, which was similar to 311-WT (**Figure 4B**). HLE variants showed a significant increase in binding affinity to human FcRn at pH 6.0 by BLI, as compared to the WT (**Figure 4C**). Based on historical success and clinical translation of LS containing mAbs, the 311-LS was selected for *in vivo* PK studies in the rhesus macaque model.

### Characterization of mAb 311-WT and 311-LS IgG

**Figure 5:**
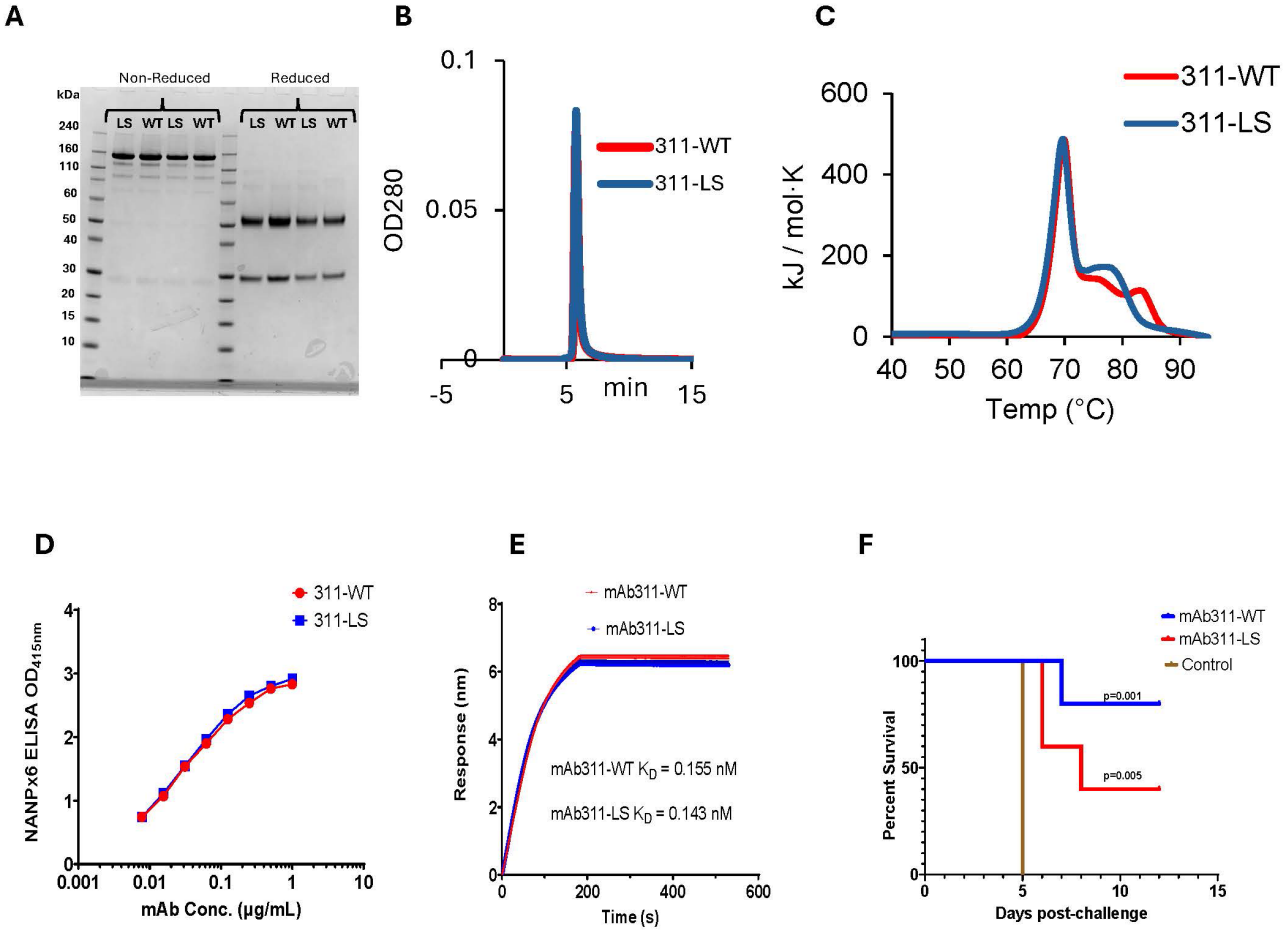
Characterization of WT and LS mAb 311. **(A)** SDS-PAGE of Protein-G purified mAb 311-LS and 311-WT IgG, analyzed under reduced and non-reduced conditions loaded in duplicate wells. **(B)** Size exclusion HPLC profile. **(C)** DSC thermogram. **(D)** ELISA reactivity to the NANPx6 peptide. **(E)** Affinity of 311-WT and 311-LS against NANPx6 peptide measured by BLI. **(F)** Survival curve of mice (*n* = 5) challenged with transgenic *P. berghei* malaria parasites following mAb 311-WT and 311-LS infusion. *p*-values are for pairwise log rank test comparing the mAbs to the control group.

MAbs were produced at a 1L-scale for *in vivo* studies. Two hundred five milligrams of 311-LS (12.5 mg/mL) and 407 mg of 311-WT (3.71 mg/mL), of similar purity, were produced as evidenced by SDS-PAGE and size exclusion chromatography (**Figures 5A, B**). MAbs contained acceptable endotoxin level <0.5 EU/mL. DSC revealed a major unfolding transition between 68 and 70 °C and a second transition between 75 and 85 °C, a melting profile typical for stable IgG molecules (**Figure 5C**). The primary melting transition was identical for the two mAbs, suggesting that the LS mutation did not significantly destabilize this folding. Compared to 311-WT that had a well-defined 82–85 °C peak, this second transition dropped off for 311-LS, indicating less stability in the CH3 region. NANPx6 peptide binding by ELISA was identical (**Figure 5D**) and both antibodies showed similar sub-nanomolar *K*_D_ by BLI (**Figure 5E**). To confirm anti-parasitic properties, 300 µg of mAbs was administered in C57BL/6 mice (*n* = 5), and the mice were challenged with 1,000 transgenic *P. berghei* sporozoites, expressing the *P. falciparum* CSP gene. Control mice were infected by day 5 and 40% of 311-LS and 80% of 311-WT transfused mice showed sterile protection. While reduced protection with the 311-LS group was concerning, the ELISA and BLI results suggested no change in antigen binding and we proceeded to compare the mAbs in the Rhesus PK model.

### Rhesus infusion

Non-human primates (NHPs) were infused with 10 mg/kg 311-LS and 311-WT IV (*n* = 2 per group, one male and one female), and sera were collected over a 28-week period for quantitation. NHPs were closely monitored for signs of immediate physical reaction, fever, redness, swelling, anaphylaxis, and cardiac and respiratory abnormalities while under sedation. As a refinement, this study sought to minimize the number of sedations, reducing invasiveness and decreasing stress and discomfort to the NHPs by acclimatizing them to a noninvasive restraint table prior to the experimental procedures of this protocol (**Supplementary Figure 4**). NHPs tolerated the table well with food enrichment and required no additional sedations outside of the initial mAb infusion, demonstrating the success of this refinement. Following mAb injection, animals were monitored for local injection site reactions, hypersensitivity, gastrointestinal symptoms, and general stress response. Blood cell counts and biochemistry data were not collected due to the blood volume required of the repeated phlebotomies needed for antibody timepoint collection. Overall, there was no evidence of adverse reaction associated with mAb-WT and mAb-LS injection in the NHPs.

### Quantitation of mAb 311 by ELISA vs. BLI

**Figure 6:**
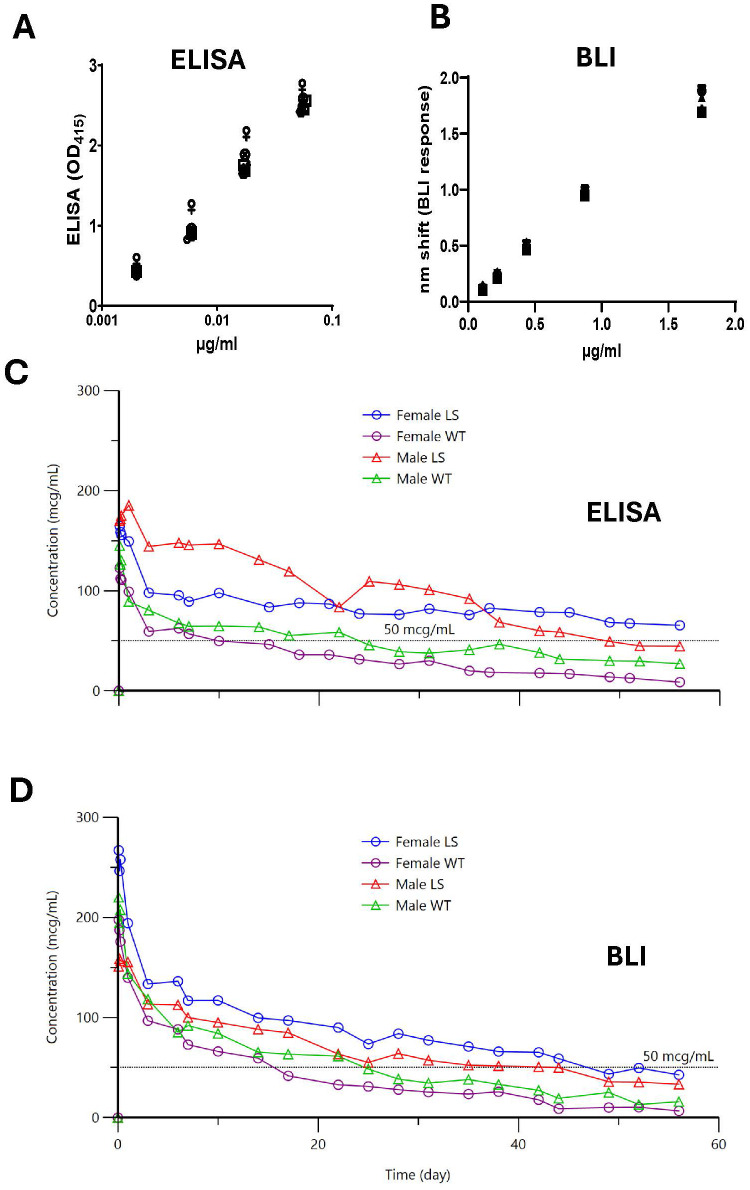
Quantitation of mAb 311-WT and 311-LS. **(A)** ELISA OD_415_ vs. antibody concentration (symbols are six independent runs). **(B)** BLI response vs. antibody concentration for nine independent runs. **(C)** Antibody concentration in individual animals up to 56 days post-infusion measured by ELISA. **(D)** Antibody concentration measured by BLI. Gray lines indicate 50 µg/mL therapeutic threshold.

ELISA is the gold standard for PK studies. In addition to allowing longer antigen–antibody interaction time, ELISA uses enzyme-labeled anti-idiotypic or anti-human antibody for detection, which makes it highly sensitive at low antibody concentrations. The ELISA protocol requires serial dilution pipetting steps, which increase assay variability. We therefore compared ELISA with a label-free BLI-based assay. BLI is more robust with higher throughput compared to ELISA because it uses commercially sourced sensors enabling mAb concentration to be determined using a single serum dilution. Standard curves for ELISA and BLI showed excellent correlation with antibody concentration (**Figures 6A, B**). After infusion, both assays showed a distribution phase followed by elimination phase (**Figures 6C, D**). The impact of HLE mutation on antibody clearance was visible, with 311-LS showing higher exposure in both assays. The serum half-life (*t*_1/2_) estimate by ELISA was greater than BLI (**Supplementary Table 1**). We demonstrate that both BLI or ELISA both be used for estimating *in vivo* mAb concentrations, but due to its higher sensitivity, ELISA data were used for the detailed PK analysis.

### PK analysis

**Figure 7:**
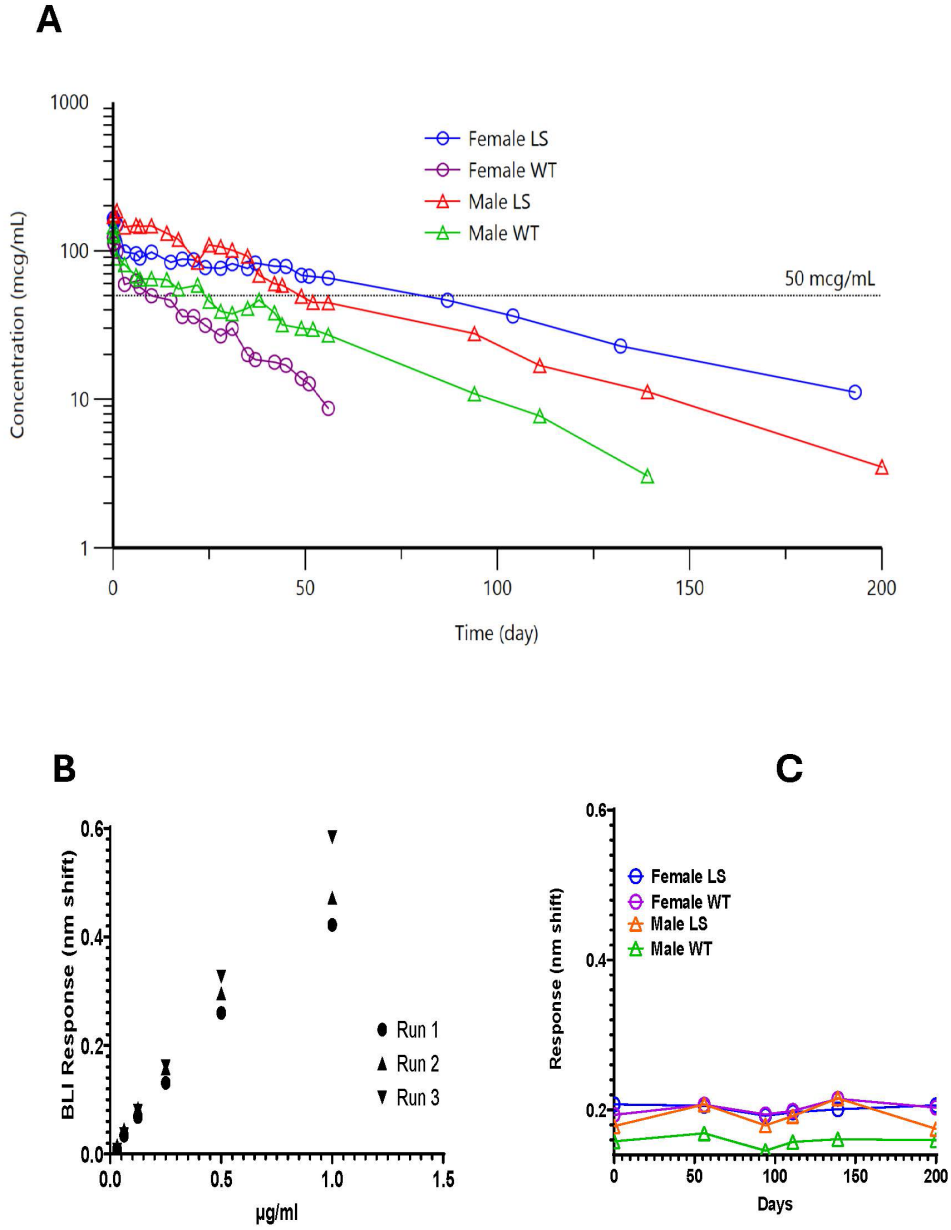
PK parameters and anti-drug antibody (ADA) detection. **(A)** Concentration of mAb 311-LS and 311-WT in individual animals during the 200 days post-infusion measured by ELISA. Concentrations greater than lower limit of quantitation were plotted; gray lines represent 50 µg/mL therapeutic threshold. **(B)** ADA assay standard curve showing reproducibility of detection of anti-Fc antibody bound to mAb-311 (positive control). **(C)** BLI detection of ADA at 1:50 serum dilution in individual animals over 200 days.

The protocol was modified to collect additional serum timepoints (up to 6 months), and ELISA was used to estimate PK parameters in individual animals (**Figure 7A**; **Table 1**). Animals used in the protocol were age-matched but the male who received the 311 LS weighed more than the others. Time-to-peak concentration, *T*_max_,was similar for both antibodies in three animals (2 h), except the larger 311-LS-infused male (24 h). Peak concentration *C*_max_ for 311-LS was higher than for 311-WT in both sexes. Based on the protective dose of CSP mAbs in human clinical trials, we assigned 50 µg/mL as the therapeutic cutoff concentration for mAb 311 (56). The mAb 311-LS cleared slower than 311-WT, as indicated by a higher therapeutic time >50 µg/mL and longer *t*_1/2_. Total exposure, as measured by *AUC*_∞_, was greater with mAb 311-LS than with 311-WT. Our study suggests that the magnitude of the distinction between the LS and WT mAb was more pronounced in females than in males. Immunogenicity of mAbs could result in neutralization and clearance (57), and we developed a BLI assay to detect rhesus antibodies against the mAb 311 (**Figure 7B**). Test sera from all 4 monkeys tested up to 200 days post-infusion showed no increase in mAb 311 binding antibody response (**Figure 7C**). Overall, 311-LS showed an improved pharmacokinetic behavior than 311-WT.

**Table 1:** Detailed PK analysis of mAb 311-LS and 311-WT using ELISA readouts.

Parameter	Female LS	Female WT	Male LS	Male WT
Animal weight (kg)	5.7	4.4	7.7	5.0
Mab dose (mg)	57	44	77	50
Rsq_adjusted	0.996	0.844	0.993	0.989
Half-life t1/2 (days)	53.5	17.6	39.3	27.8
Tmax (h)	2	2	24	2
Cmax (µg/mL)	164.7	122.73	185.2	145.1
AUC∞ (day*µg/mL)	9771	2107	8191	3875
AUC_%Extrap_pred (%)	8.76	11.96	2.55	3.53
Vz_F_pred (mL)	455.3	530.9	533.4	520.8
CL_pred (mL/day)	5.89	20.87	9.4	12.95
Time > 50 µg/mL (days)	80.2	9.8	48.6	23.8

## Discussion

A clinical malaria candidate mAb CIS43, included as a reference specificity control in the TCR assay, revealed no human TCR. Since mAb CIS43 has a track record of safety in humans, this control was important to objectively compare with the observed immunological cross-reactions of mAbs 311 and 317. Therapeutic mAbs often show some level of poly-specificity, as evidenced from a membrane proteome array study that found 32% of 250 preclinical candidates having some degree of off-target binding (58). We observed no immuno-reactions with cell membranes of 14 human tissues for either mAb 311 or 317, which was an encouraging finding. Although cytoplasmic reactivity was observed for both mAb 311 and 317, there is limited ability of antibody drugs to access the cytoplasmic compartment *in vivo*, and the weak and sporadic cytoplasmic binding of mAbs 311 and 317 were considered of little to no toxicologic significance (37, 59). Immunoreactivity of mAb 317 to extracellular elements in the glomeruli was more concerning as large biomolecules are filtered by the kidneys (60–62). While toxicologic significance of extracellular binding is unknown, immune complexes in the kidney have been implicated in glomerulonephritis and nephrotoxicity (63, 64). The testis is generally considered an immune-privileged site. Thus, the weak reactivity of mAb 311 to extracellular elements of the testis and intestine was less concerning than mAb 317 reactivity in the kidneys. Overall, we show no membrane reactivity with any of the CSP mAbs, a negative TCR profile for the reference mAb CIS43, and weak, but positive extracellular and cytoplasmic cross-reactions for the two prototype CSP mAbs 311 and 317. These data highlight the importance of *in vitro* TCR assays to look for off-target binding early during the mAb development cycle.

Mab 317 cross-binding has been reported by others (30, 31), and there can be several reasons for the observed poly-specificity (1). Non-specific binding to “sticky” fixed tissue material (2). While no significant sequence similarity with mAb 317 epitope (NPNA)_3_ is known in human proteins, mAb 317 requires only NPN residues to bind with high affinity and cross-binding to DPN motifs is known (54) (3). CSP repeats are unstructured and mAb binding stabilizes these repeats into type I β-turn motifs. Thus, the observed cross-reactivity could be due to CSP repeat-like binding motifs formed by unrelated human proteins (4). CSP Fabs form super helical complexes stabilized by inter-Fab homotypic contacts, which could also lead to off-target binding (29, 65, 66). Despite its high affinity and superior preclinical protective efficacy, mAb 317 TCR data revealed potential off-target liabilities that precluded its clinical progression.

Prophylactic mAbs against infectious diseases like *Staphylococcus aureus* (67, 68), malaria (47), RSV (10, 11), and SARS-CoV-2 (12, 13) are required to circulate above therapeutic concentration for several months. The LS mutation has been successfully used to extend the half-life of malaria mAb CIS43 (21), L9 (26), and MAM01 (31) in clinical settings. Here, 311-LS showed similar antigen binding by ELISA and BLI but reduced protection in the mouse model as compared to 311-WT. While intravenous parasite challenge and sterile protection are a stringent test of mAb function, the observed reduction in mouse protection warrants further evaluation. Indeed, HLE mutations are known to alter Fc structure (69), and IgG effector function has been implicated in protection against malaria (70). Our data suggest that multiple HLE strategies may need to be evaluated preclinically to best preserve Fab and Fc function of the parent molecule.

Factoring in the historical success of LS mutation, we advanced 311-WT and 311-LS to the rhesus PK model. While transgenic FcRn mouse models are available, the conservation of human and NHP FcRn makes rhesus the most reliable model for translational studies. In NHPs, 311-LS and 311-WT were well tolerated with no sign of anaphylaxis, local injection site reaction, or weight change at the 10 mg/kg dose. LS mutation improved FcRNn binding affinity ~10-fold. In rhesus, a 41% and 204% improvement in *t*_1/2_ and a 104% and 713% improvement in therapeutic time (>50 µg/mL) were observed in the male and female, respectively. CIS43LS mutation tested in rhesus improved FcRn binding affinity 9- to 13-fold and *t*_1/2_ by 77% (47). A limitation of our rhesus PK study was that we administered one infusion at a single antibody dose, via one route of administration in two animals per group. The follow-up period was relatively short and certain PK parameters could not be estimated with a high degree of confidence. Nonetheless, the observed alignment between our data and existing literature underscores the reliability of the rhesus PK model for advancing mAb-based interventions. We also show that a more robust and less labor-intensive BLI assay can be an alternative to ELISA for PK studies. We note that females showed a greater increase in *t*_1/2_ of 311-LS, which is similar to previous reports (71). Understanding the impact of sex, weight, and age on PK could help optimize dosing strategies and enhance therapeutic efficacy across diverse populations. A single dose administration of extended half-life malaria mAb can be a critical tool to confer prophylactic protection among travelers and seasonal protection in Africa.

## Data Availability

The original contributions presented in the study are included in the article/**Supplementary Material**. Further inquiries can be directed to the corresponding author.

## Author disclaimer

The views, opinions and/or findings contained in this presentation are those of the author and do not necessarily reflect the views of the Department of Defense and should not be construed as an official DoD/Army position, policy or decision unless so designated by other documentation. Reference herein to any specific commercial products, process, or service by trade name, trademark, manufacturer, or otherwise, does not necessarily constitute or imply its endorsement, recommendation, or favoring by the U.S. Government.
