# Supplementary figures and images for: Comparative assessment of tissue cross-reactivity and pharmacokinetic half-life of malaria monoclonal antibodies
Source: Front Immunol. 2026 Jun 23;17:1848315. doi: 10.3389/fimmu.2026.1848315 (PMC13337405; doi:10.3389/fimmu.2026.1848315)

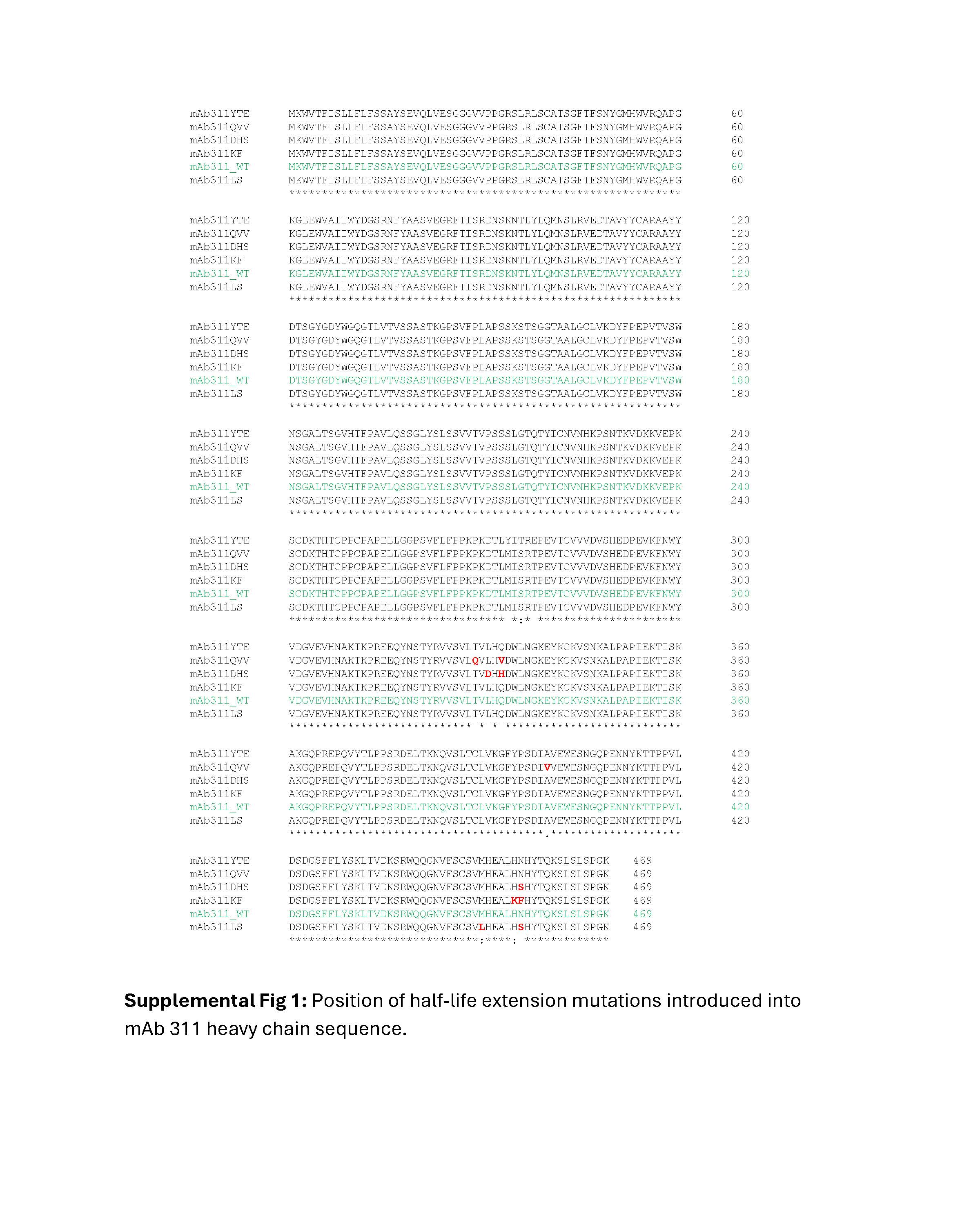

Supplement: Supplementary file 1 [file Image1.jpeg]

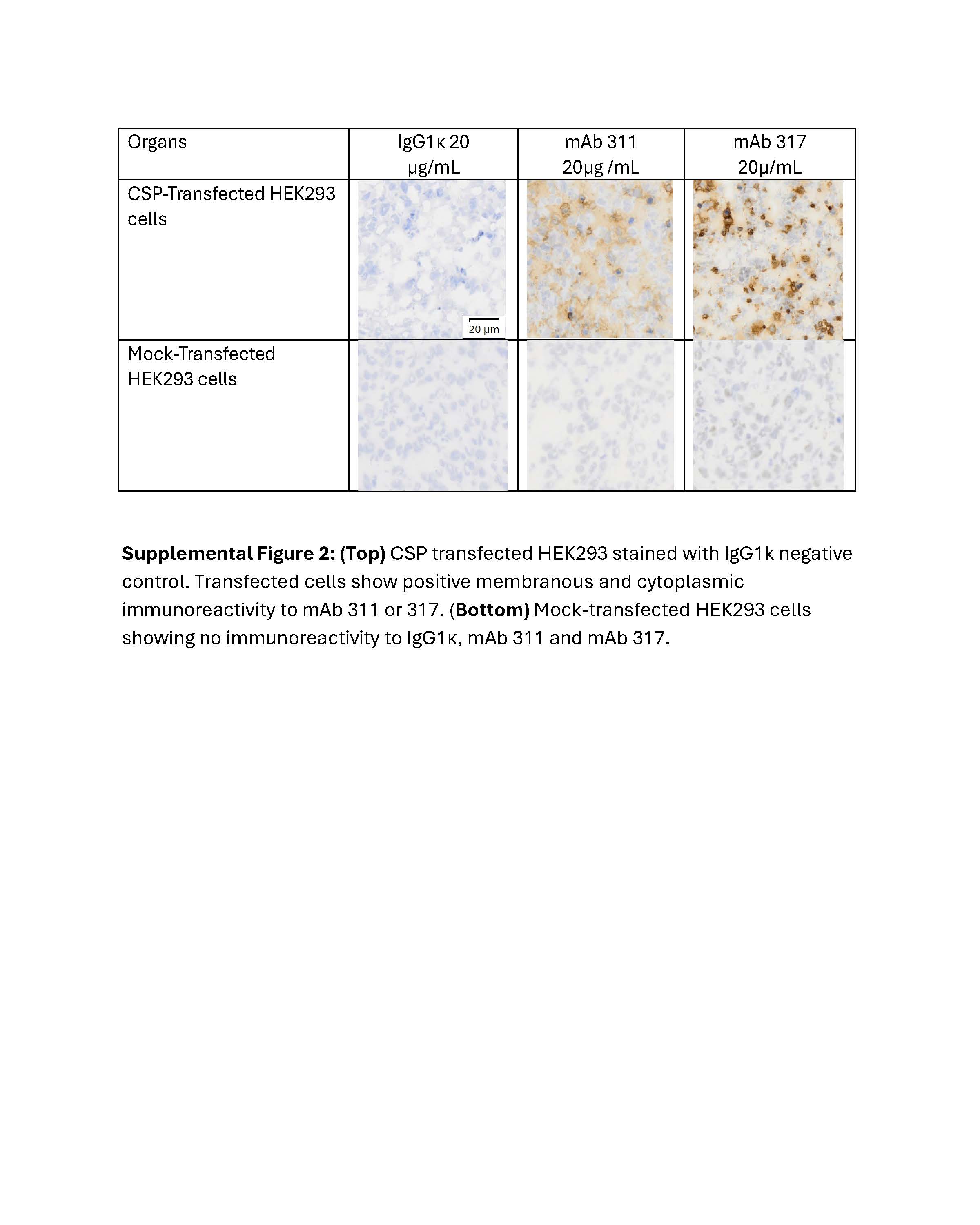

Supplement: Supplementary file 2 [file Image2.jpeg]

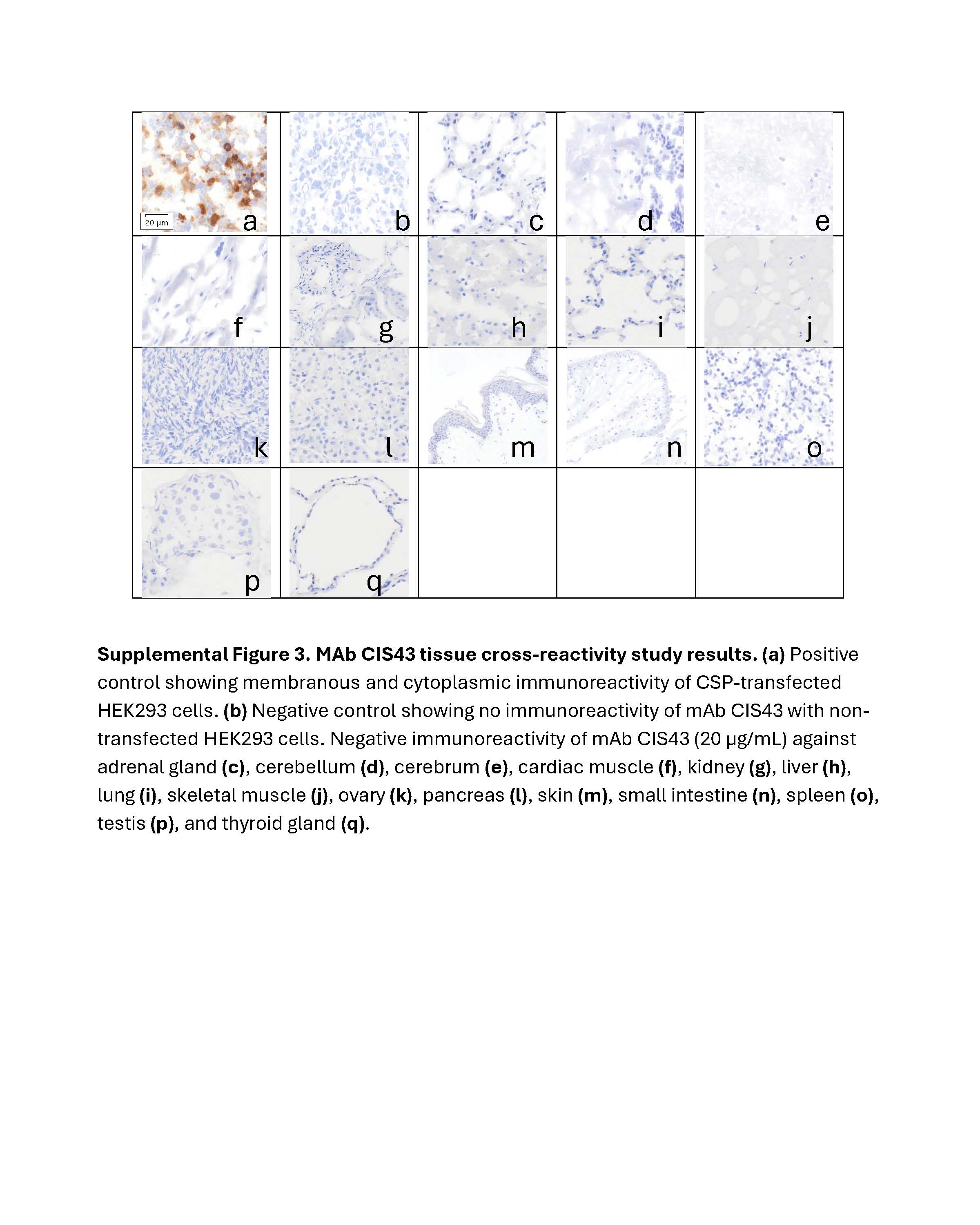

Supplement: Supplementary file 3 [file Image3.jpeg]

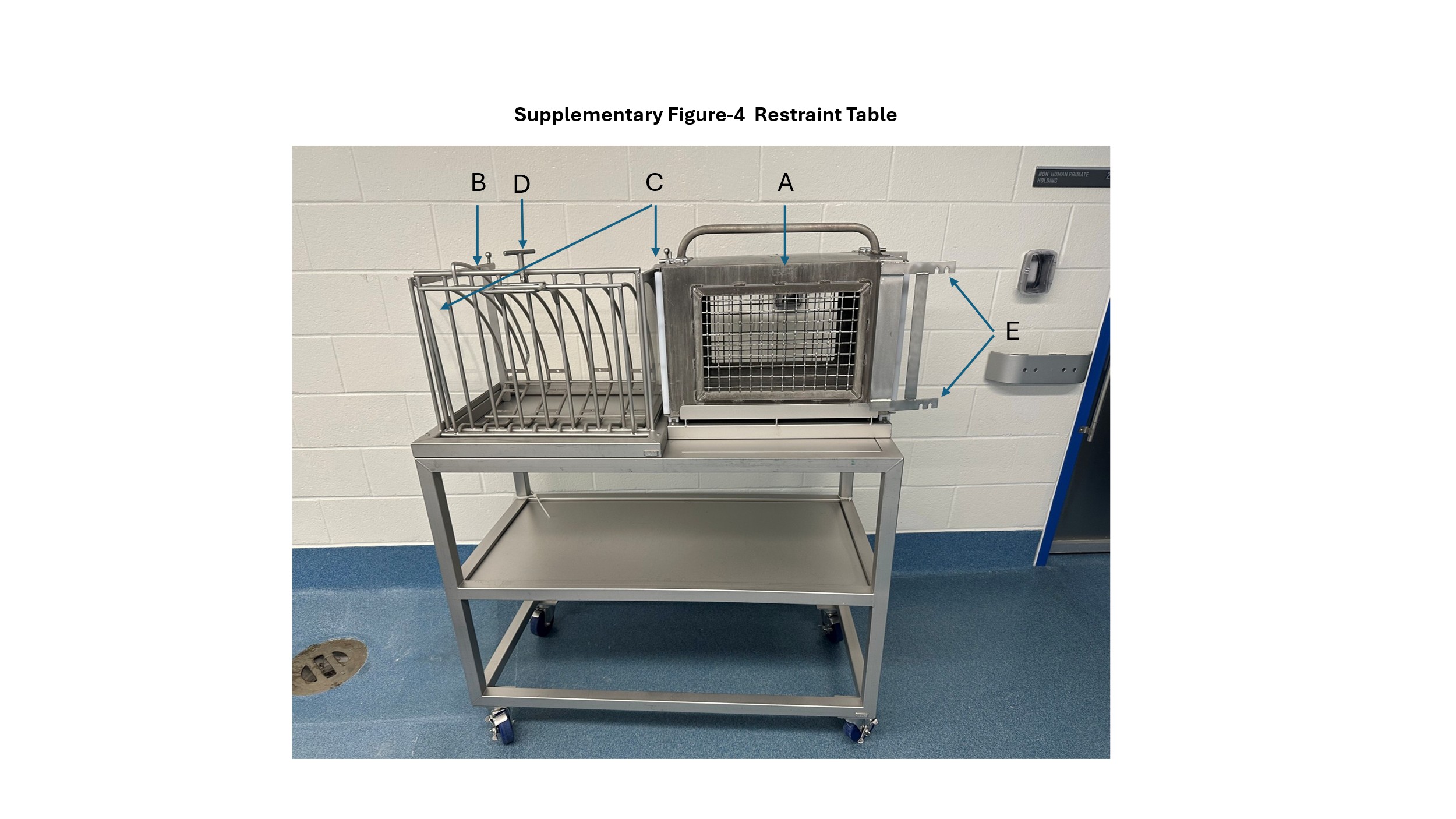

Supplement: Supplementary file 4 [file Image4.jpg]

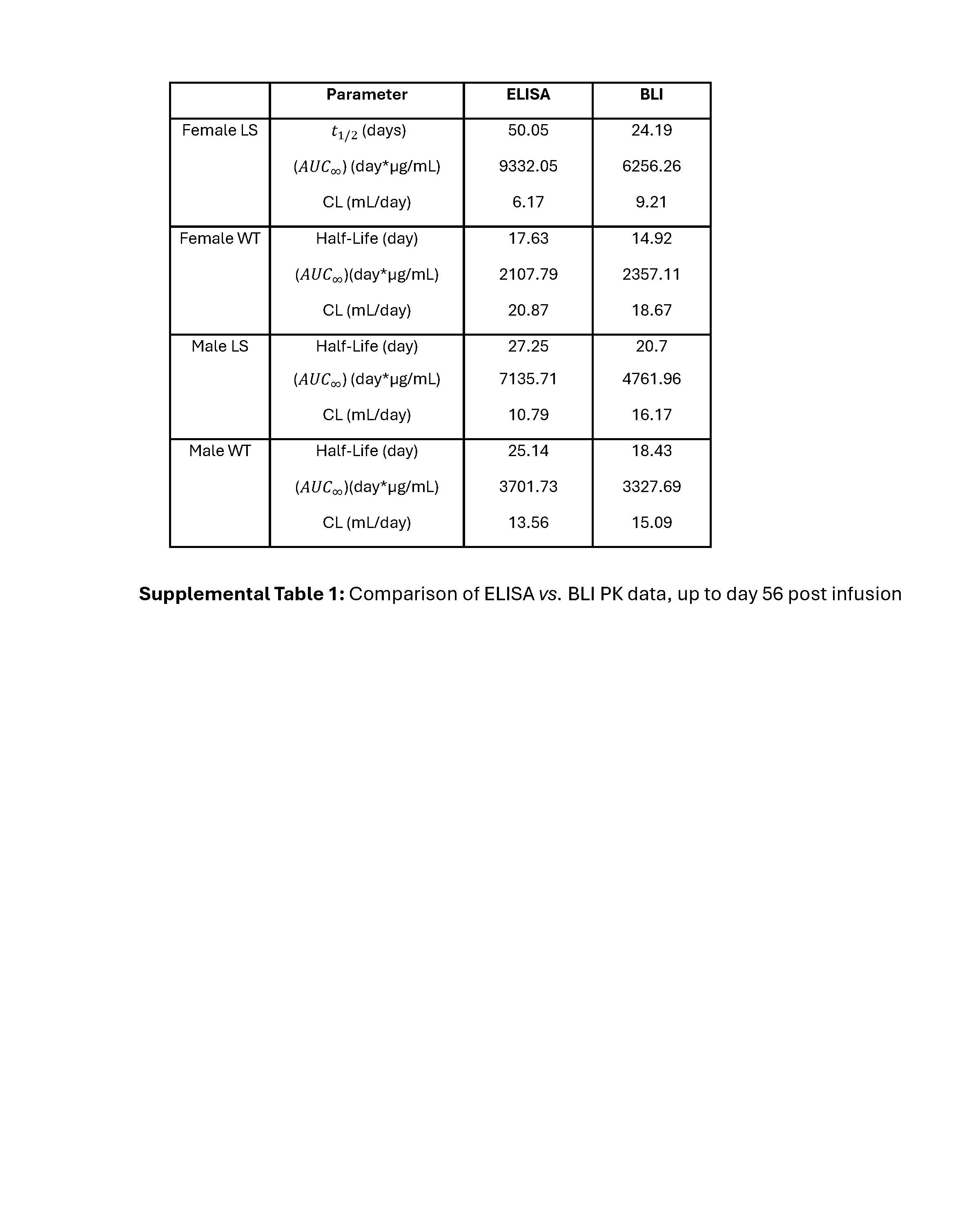

Supplement: Supplementary file 5 [file Image5.jpeg]
